# Development of a human leukocyte antigen-based HIV vaccine

**DOI:** 10.12688/f1000research.13759.1

**Published:** 2018-06-22

**Authors:** Yufei Wang

**Affiliations:** 1 Mucosal Immunology Unit, Dental Institute, Kings College London, Guy’s Campus, London Bridge, London, SE1 9RT, UK

**Keywords:** HIV vaccines, HLA, allogeneic immune response

## Abstract

Human immunodeficiency virus (HIV) carries abundant human cell proteins, particularly human leukocyte antigen (HLA) molecules when the virus leaves host cells. Immunization in macaques with HLAs protects the animals from simian immunodeficiency virus infection. This finding offers an alternative approach to the development of HLA molecule-based HIV vaccines. Decades of studies have enhanced a great deal of our understanding of the mechanisms of allo-immune response-mediated anti-HIV immunity. These include cell-mediated immunity, innate immunity, and antibody response. These studies provided a rationale for the future design of effective HIV vaccines.

## Introduction

Vaccination is the most efficient and cost-effective way to prevent infectious diseases, which has been proven in diseases such as smallpox, polio, and yellow fever. However, despite decades of research, development of an effective human immunodeficiency virus (HIV) vaccine has not been successful. Currently, there are an estimated 37 million HIV-infected people worldwide, and only half of them are receiving anti-retroviral therapy (ART). It is unlikely that ART alone will stop all HIV infections and end the epidemic, and an HIV vaccine is essential for ending the HIV/AIDS pandemic ^1^.

The concept of an allo-immunization vaccine strategy was conceived decades ago ^2– 4^. In 1991, James Stott and colleagues first demonstrated that macaques immunized with human T-cell lines alone exhibited sterilizing immunity to intravenous challenge with simian immunodeficiency virus (SIV) grown in the same T-cell lines ^5^. Subsequent studies identified that human leukocyte antigen (HLA) molecules expressed on the T-cell lines play an important role in eliciting protection ^6^. These findings raised the possibility that HLAs in humans (allo-immune reactions) can be used in HIV vaccines, as HLA genes are highly polymorphic in the human population and induce potent cellular and humoral immune responses. This idea of potentially using HLA as constituents in HIV vaccines has been advocated by a group led by Thomas Lehner in over a decade’s research, which gained further insight into the mechanisms of allo-immunity-mediated protection ^3, 4^. These include antibodies, cell immunity, and innate immunity. The use of purified recombinant HLA class I and II alleles combined with viral antigens has demonstrated the significant role of HLA molecules and viral antigens in eliciting protection in macaques ^7^. Similar studies were carried out in allo-immunization in macaques by using recombinant Mamu major histocompatibility complex (MHC) molecules combined with SIV antigens ^8^.

On its surface, HIV displays envelope (Env) glycoproteins (termed spikes) that are composed of two subunits: three molecules of gp120 linked non-covalently to three molecules of gp41. Between the viral envelope proteins, the viral lipid membrane contains abundant host cell-derived HLAs as well as some other host-cell proteins which are selectively incorporated into the envelope of the virions. One study estimated that more HLA proteins than gp140 molecules are incorporated into the membrane of virions ^9^. HLA molecules have both well-defined and stable polymorphisms, making them an attractive alternative target for HIV vaccine design.

This review will summarize previous studies on HLA-based HIV vaccines and some recent new findings. It will also discuss the future designs of HLA allo-antigen-based HIV vaccines.

## HLA polymorphism

HLA class I molecules (A, B, and C) have over 12,000 different alleles and class II molecules have over 4,000 alleles among populations. HLA class I and II molecules are heterodimeric and have variable extracellular and relatively constant transmembrane and intracytoplasmic domains. The class I molecule consists of a 45 kDa heavy chain and a light chain (β-2 microglobulin), and the MHC class II molecule is composed of two 30 kDa membrane-spanning proteins. HLA genes contain eight exons. Exons 2 and 3 encode the α-1 and α-2 domains for class I and the α-1 and β-1 for class II, which both bind and present the peptide to T cells. The great majority of the polymorphism found in the class I and II genes occurs in the α-1 and α-2 (class I) and the α-1 and β-1 (class II) domains ^10^.

Allogeneic non-self-antigens are responsible for inducing allo-immune reaction. HLAs are the major allo-antigens. These highly polymorphic antigens, which are expressed on all nucleated cells, are capable of eliciting unusually large polyclonal T-cell responses and antibodies. Two forms of allo-recognition of foreign HLA molecules exist. The first involves recognition by T cells of intact non-self-MHC molecules complexed to endogenous peptides (“direct” allo-recognition). The second pathway involves the recognition of allogeneic MHC molecules as peptides presented by self-MHC molecules (“indirect” allo-recognition). Allo-recognition is characterized by uniquely high frequencies of responding T cells. The mechanism of allo-recognition is believed to be part of the self-restricted T-cell repertoire established by “positive selection”. Direct alloreactivity is the result of cross-reactivity of T-cell receptors (TCRs) that bind self-HLA-restricted peptides. However, recognition of MHC allo-antigens by allo-specific B cells is quite different from T cells. Allo-antigen polymorphic determinants are readily bound by B-cell receptors (surface immunoglobulins), which can be internalized and present as HLA-peptide antigen to T cells. This represents an important route of amplifying immune response and also producing anti-HLA antibodies ^11^.

## Cell-mediated anti-HIV allo-immunity

T-cell response to allogeneic HLA molecules represents a powerful natural immune response. Development of allogeneic responses seems to be not uncommon among the human population, and this has been described in heterosexual and homosexual monogamous partners practicing unprotected sex, which showed allogeneic CD4 ^+^ and CD8 ^+^ T-cell proliferative responses to the partners’ unmatched cells ^12, 13^. CD4 ^+^ T cells from these allogeneic responders (recipients) also showed resistance to HIV infection *in vitro*, suggesting that cell-mediated allogeneic response may play an important role in the prevention of HIV infection ^12^. Genetic, epidemiological, and experimental evidence showed that the HLA molecules are critical in controlling HIV infection ^14^. Sharing HLA class I molecules, particularly HLA-B alleles, is associated with an increased risk of HIV-1 transmission in discordant couples and HIV vertical transmission between mother and child ^15– 17^. These findings suggest that allogeneic immune responses elicited by HLA-B may play an important role in the protection against HIV transmission. Early studies demonstrated that both systemic allo-immunization in humans and mucosal allo-immunization in macaques significantly upregulated the concentrations of CD8 cell-derived soluble anti-HIV factors, such as HIV suppressor factor, CCL2, 3, and 5, which also downregulated the proportion of cells expressing CCR5 and CXCR4, the co-receptors for HIV infection. These *in vivo* allogeneic stimulated CD4 ^+^ T cells also showed a dose-dependent decrease in HIV/SIV infectivity *in vitro* ^18, 19^. In addition to producing anti-HIV soluble factors, allogeneic CD8 ^+^ T-cell responses were reported to be able to kill incoming infected CD4 ^+^ cells which may carry viral antigen-associated HLA class I molecules and therefore may play a complementary role in reducing cell-associated transmission ^20^. It is interesting to note that only HLA-B-mediated allogeneic cytotoxic T lymphocyte (CTL) responses can lead to significant cell killing ^20^. The reason for this is not clear; it may be because of higher precursor frequency against HLA-B alleles than HLA-A alleles observed in the human population ^21, 22^.

## Innate immunity

Innate immunity is an early response system, largely independent of prior encounter with a pathogen. Innate immunity can be classified into cellular, extracellular, and intracellular components. The human immune system has developed a number of innate mechanisms that can interrupt HIV infection at various stages: (i) inhibition of HIV-1 by downmodulation or blocking of the CCR5 co-receptor induced by an increase in the CC chemokines CCL3, CCL4, and CCL5 and (ii) inhibition of HIV-1 which may have escaped the CCR5-mediated mechanism by upregulation of intracellular host-encoded HIV-1 restriction factors by interfering with viral RNA reverse transcription and post-integration restriction and adherence. In recent years, a number of restriction factors of HIV replication have been described, such as APOBEC3G (A3G, apolipoprotein B mRNA editing enzyme, catalytic polypeptide-like 3G) or F protein, TRIM5-α, Tetherin, SAMHD, and MX2 ^23^.

Stimulation of human CD4 ^+^ T cells with allogeneic cells or recombinant HLA-A construct *in vitro* upregulated A3G mRNA, which is correlated with the allogeneic T-cell proliferative responses ^24, 25^. The mechanism of upregulation of A3G mRNA involves interaction between HLA on dendritic cells (DCs) and TCR of CD4 ^+^ T cells, which is ZAP70 and downstream ERK phosphokinase signaling dependent and induces CD40L and A3G mRNA expression in CD4 ^+^ T cells ^25^. Allo-immune response-induced A3G was found to be significantly increased in CD4 ^+^CD45RA ^+^ naive, CCR5 ^+^ and CD45RA ^-^CCR7 ^−^ effector memory T cells ^25^. *In vivo* studies of women allo-immunized with their partners’ peripheral blood mononuclear cells also showed a significant increase in A3G protein in CD45RO ^+^ memory and CCR7 ^−^ effector memory T cells. The functional effect of allo-stimulation upregulating A3G was demonstrated by a significant decrease in *in vitro* infectivity ^25^.

Systemic immunization of rhesus macaques with recombinant HLA constructs, linked with HIV/SIV antigens and heat shock protein 70 (HSP70) to dextran, showed significant upregulation of A3G in CD27 ^+^ memory B cells and CD4 ^+^ effector memory T cells ^26^. Interestingly, activation-induced cytidine deaminase (AID), a member of the deaminase family, is also upregulated. AID is important for antibody somatic hypermutation and class switch recombination, and upregulation of AID in B cells is directly correlated with A3G in B and T cells, and both AID and A3G upregulation was correlated with protection against SHIV (simian/human immunodeficiency virus) challenge in macaques ^26^. There was also an increase in interleukin-15 (IL-15) in DCs and CD40L in CD4 ^+^ T cells. IL-15 binds the IL-15 receptor complex in CD4 ^+^ T and B cells and upregulates A3G, which can be further enhanced by CD40L–CD40 interaction.

## The role of antibodies

The role of anti-cell antibodies, particularly anti-HLA antibodies, in protection against SIV/HIV infection has been studied extensively. Numerous studies have demonstrated that antibodies to HLA molecules can effectively neutralize HIV-1 in a complement-dependent manner ^5, 6, 27– 29^. These studies also shed some light on the mechanisms of anti-HLA antibodies produced in macaques (xeno) and in humans (allo) in protection against SIV/HIV infection and the importance of adequate antibody titers and adjuvant used.

In xeno-immunization of macaques with human T-cell lines, the induction of anti-HLA antibodies plays an important role in protection ^5– 7^. Recent studies using recombinant HLA class I and II and HIV/SIV antigens demonstrated that anti-HLA antibody alone is not sufficient in eliciting protective immunity against heterologous SHIV challenge in rhesus macaques. The protection was achieved in combination with viral antigens and was able to be passively transferred by serum ^7^. There is evidence that allo-antibodies can also protect against HIV/SIV infection ^8^. Immunization of macaques with recombinant Mamu MHC constructs and HIV gp120 elicits plasma and mucosal IgG and IgA antibodies to the antigens and protects against rectal challenge with SHIV. In humans, induction of allo-antibodies has been demonstrated in women receiving whole-cell allo-immunization in the form of leukocyte immunotherapy for recurrent spontaneous abortion ^10^. The role of anti-HLA antibodies in protection against HIV infection *in vivo* is not clear; *in vitro* studies suggest that the antibodies can neutralize HIV-1 infection in cell-based assay ^27^. Both anti-HLA antibodies induced in macaques (xeno) and humans (allo) neutralize SIV/HIV grown in the donor CD4 ^+^ T cells in a complement-dependent manner ^7, 27– 29^.

The binding epitopes of polyclonal anti-HLA antibodies induced in macaques (xeno) and in humans (allo) are significantly different. It has been shown using HLA bead arrays that macaque anti-HLA antibodies were directed against whole HLA structure (polymorphisms and non-polymorphism determinants) and bound to almost all HLA alleles irrespective of the HLA alleles used for immunization, and this was demonstrated in cell line-immunized ^29^ and purified HLA molecule-immunized macaques ^7^. In contrast, polyclonal allo-antibodies produced in humans, such as transplant patients ^30^ and multiparous women ^31^, are usually directed against HLA polymorphism. Furthermore, allo-antibodies induced in allo-immunized women are demonstrated to be specific to HLA molecules present in the donor haplotype but not recipient haplotypes ^28^. This difference may explain the different efficacy between xeno- and allo-immunization in protection against SIV/HIV infection.

## Other antibodies

Allo-immunization with unmatched leukocytes from partners of women with recurrent spontaneous abortion elicits specific antibodies to the CCR5, the co-receptor for R5 HIV. These antibodies were also found in the sera of multiparous women who were naturally immunized by semi-allogeneic fetal antigens. Antibodies to CCR5 have been isolated from healthy donors, in CCR5-lacking subjects (Delta32 mutation) who were sensitized with CCR5 ^+^ cells, in HIV-infected patients, and from HIV-exposed, seronegative (ESN) subjects ^32^. Antibodies to CCR5 were also found in rhesus macaques immunized with SIV grown in human CD4 ^+^ T cells ^33^ and allo-immunized women ^34^. CCR5 antibodies were also demonstrated in macaques immunized with various domains of CCR5 molecules and significantly protect macaques from SIV infection ^35, 36^. Recent studies found that administration of antibodies to the integrin α4β7, the homing receptor, leads to significant protection from transmission ^37^. Whereas antibodies to CCR5 can inhibit R5 HIV entry by blocking CCR5 on the cell surface, the antibodies against α4β7 incorporated into the envelope of HIV-1 virions ^38^ may play an important role in protection.

## The prospect of an HLA molecule-based vaccine

Most research in developing HIV vaccines has been focused on the induction of protective immune responses against HIV-encoded proteins. It is well known that HIV uses several strategies to evade the host immune system. A high degree of glycosylation in combination with an error-prone reverse transcriptase leads to conformational masking of conserved epitopes. High mutation rates not only make it difficult for the immune system to generate broadly neutralizing antibodies against HIV-1 Env but also enable the virus to escape the CD8 ^+^ T-cell-mediated CTL response. The abundant host membrane proteins, particularly HLA molecules that can elicit protective immunity in macaques, present an alternative approach to developing effective HIV vaccines ^2– 4, 39, 40^.

There are a number of obstacles to be overcome in designing HLA molecule-based vaccines. It is evident that an HLA molecule as a xeno-antigen is more effective in macaques to protect against SIV or SHIV infection. Emerging evidence shows that immune response to HLA non-polymorphic regions may play an important role in eliciting protection, as was demonstrated in xeno-immunized macaques ^7, 29^. In contrast, in allo-immunized humans, immune response is largely directed against HLA molecule polymorphism ^28– 31^. The importance of immune responses to non-polymorphic epitopes in protection against HIV infection can also be envisaged, given that HIV carries a donor’s HLA only in the initial exposure to the virus when the recipient’s allo-immune response may have the best opportunity to prevent infection. Once the virus has established infection in the individual host, the viral envelope will carry recipient HLA molecules and the allogeneic-based immune response directed to the polymorphism may diminish its effect to eliminate the virus. To induce immune response to HLA non-polymorphic epitopes, which are effectively auto-antigens, one needs to break down immune tolerance. This can be achieved by using a different immunization regime or by inducing cross-reactions to HLA non-polymorphic determinants through the modification of antigens. However, if one induced immune response to HLA non-polymorphism, the risk for the host is not clear, as some antibodies against T-cell surface molecules can cause severe adverse effects ^41^. An effective HLA antigen-based vaccine also needs to be able to elicit high titers of antibodies, which were demonstrated to be correlated with HIV-neutralizing activity *in vitro* and the viral load in the infected macaques *in vivo* ^7^. In order to achieve a high titer of antibodies, particularly in humans of allo-immunization, one needs to consider using more potent adjuvants, such as TLR agonist HSP70 ^7^.

A vaccine including only HLA molecules may not be sufficient to elicit protection ^7^. Other antigen components, such as viral antigens, may be required in order to achieve protection ^7^. The reason for this is not clear. Studies have shown that antiviral activity of anti-HLA allo-antibodies can be greatly enhanced in combination with anti-HIV protein antibodies ^28^. Heteroligation of HIV gp140 and non-HIV antigens greatly increases the binding affinity of antibodies, and many conventional anti-HIV-neutralizing antibodies cross-react with cell-associated antigens, which may contribute to the neutralizing effect ^42^. Furthermore, multiple components likely increase vaccine immunogenicity to elicit humoral, cellular, and innate immune responses.

## Conclusions

Since it was discovered that xeno-immunization response induced by human T-cell lines can protect macaques from SIV infection, a great deal of research has been carried out on the possibility of using human cell antigens, in particular HLA molecules, as allo-antigen-based HIV vaccines. The polymorphic nature of HLA molecules and the potency of allogeneic immune reaction have been demonstrated to elicit anti-HLA antibodies, T-cell-mediated anti-HIV immunity, and innate anti-HIV immunity that can neutralize HIV, inhibit HIV infection by blocking viral entry or post-entry transcription, or eliminate virus-infected cells. Studies also show that immune response to HLA non-polymorphic determinants may be important in protection against HIV infection. Harnessing this potent force of allogeneic immune responses could benefit our design of HLA molecule-based HIV vaccines which can play an important role in combating the HIV pandemic.
